# Exposure–outcome overlap in the AKI–AKD–CKD continuum: a comment on Hu et al.

**DOI:** 10.1080/0886022X.2026.2757320

**Published:** 2026-11-08

**Authors:** Abuzar Ali

**Affiliations:** Pak Red Crescent Medical and Dental College, Lahore, Pakistan

To the editor,

I read with interest the study by Hu et al. on acute kidney disease (AKD) after acute kidney injury (AKI) [1]. Its large cohort and use of the Acute Disease Quality Initiative (ADQI) 16 staging framework provide useful evidence on kidney recovery [2]. Three points may help clarify the reported association with *de novo* chronic kidney disease (CKD).

First, the exposure and outcome may partly capture the same persistent dysfunction. AKD staging used the highest serum creatinine from day 7 to before day 90. CKD follow-up began at day 90. The authors state that CKD required kidney abnormalities lasting at least three months. However, the number and timing of measurements used to confirm chronicity are unclear. Sustained creatinine elevation can contribute to both classifications. The adjusted hazard ratio (HR) of 1.92 (95% confidence interval [CI], 1.70–2.17) may therefore reflect continuity as well as later disease development. This does not invalidate AKD as a prognostic marker. Reporting CKD present at day 90 separately from later diagnoses would help. A later landmark analysis among survivors still free of CKD could also test the association with subsequent events.

Second, the stage 0 C estimate increased after adjustment. Compared with stage 0 A/0B, the crude HR was 1.14 (95% CI, 0.92–1.40), whereas the adjusted HR was 1.74 (95% CI, 1.41–2.15). Models that add baseline estimated glomerular filtration rate and peak AKI stage in separate steps could clarify this change. The shift alone does not establish model bias. The reference group also needs careful interpretation. The study assigned stage 0 A/0B when follow-up creatinine was below baseline. A low creatinine value may reflect fluid dilution or reduced muscle mass, rather than complete kidney recovery [2]. These explanations remain untested in this cohort.

Third, mortality and follow-up selection deserve separate consideration. The Fine–Gray analysis addressed deaths within seven days in the AKI-to-AKD analysis [3]. For the CKD analysis, please clarify how deaths before day 90 affected eligibility and how later deaths were handled. If later deaths were censored, the Cox estimates should be interpreted as cause-specific hazards. A cumulative incidence analysis accounting for death would address absolute CKD risk. Creatinine data for CKD assessment were missing for 11,592 of 18,189 patients without documented prior CKD (63.7%). The authors acknowledge this limitation and compare included and excluded patients. If feasible, inverse-probability weighting for follow-up availability could test sensitivity to selection on measured characteristics. It would not resolve bias from unmeasured causes of missingness.

I support the authors’ call for structured follow-up after AKI. These clarifications would help readers judge the strength and scope of the findings.

## Data Availability

No new data were generated or analyzed for this letter. The discussion is based on the published sources cited in the references.
